# ‘*The company is using the credibility of our profession*’: exploring experiences and perspectives of registered dietitians from Canada about their interactions with commercial actors using semi-structured interviews

**DOI:** 10.1017/S1368980024001733

**Published:** 2024-10-04

**Authors:** Virginie Hamel, Mélissa Mialon, Jean-Claude Moubarac

**Affiliations:** 1 Centre de Recherche en Santé Publique, Department of Nutrition, Faculty of Medicine, University of Montreal, Montreal, QC H3C 3J7, Canada; 2 French School of Public Health (EHESP), CNRS UMR 6051 Arènes/Inserm U1309 RSMS, Rennes, France

**Keywords:** Corporate political activity, Registered dietitians, Commercial actors, Professional practice

## Abstract

**Objective:**

To gain insight into the experiences and perspectives of registered dietitians (RD) in Canada regarding their interactions with commercial actors and actions undertaken to manage these interactions.

**Design:**

Qualitative study using semi-structured interviews combined with a document analysis.

**Setting:**

Quebec, Canada

**Participants:**

RD aged ≥ 18 years (*n* 18)

**Results:**

All participants reported interacting with commercial actors during their careers, such as receiving continuing education provided or sponsored by food companies. RD in Quebec perceive these interactions as either trivial or acceptable, depending on the commercial actor or interaction type. Participants discussed how certain interactions could represent a threat to the credibility and public trust in dietitians, among other risks. They also discussed the benefits of these interactions, such as the possibility for professionals to improve the food supply and public health by sharing their knowledge and expertise. Participants reported ten mechanisms used to manage interactions with commercial actors, such as following a code of ethics (individual level) and policies such as partnerships policy (institutional level). Finally, RD also stressed the need for training and more explicit and specific tools for managing interactions with commercial actors.

**Conclusions:**

RD in Quebec, Canada, may engage with commercial actors in their profession and hold nuanced perspectives on this matter. While some measures are in place to regulate these interactions, they are neither standardised nor evaluated for their effectiveness. To maintain the public’s trust in RD, promoting awareness and developing training on this issue is essential.

Over the last decades, financial relationships between commercial actors and health professionals have raised concerns about conflict of interest (COI), which arises ‘whenever activities or relationships compromise the loyalty or independent judgment of an individual who is obligated to serve a party or perform certain roles’^(1)^. Interactions between commercial actors such as food manufacturers or multinational food companies and nutrition professionals like registered dietitians (RD) have been discussed and scrutinised in countries including the USA^(2,3)^ and Australia^(4)^. COI arising from relationships between nutrition professionals and the food industry can lead to harms such as damaging the trust, credibility, integrity and reputation of RD and their professional body, as well as misleading the public about nutrition knowledge^(5)^. This influence is part of a spectrum of practices the food industry uses to ‘secure preferential treatment and/or prevent, shape, circumvent or undermine public policies in ways that further corporate interests’, known as corporate political activity^(6)^.

Several studies have revealed how corporate political activity impacts public health policy, research and practice on healthy diets^(7,8)^. However, very few empirical studies have examined, more specifically, how corporate political activity may influence the work of health professionals, such as RD, who have an important role in protecting and promoting public health through their individual or population-based interventions^(9,10)^. In other fields, such as medicine, the pharmaceutical industry’s influence has already been well studied and criticised over the last decades. Measured influences such as the positive association between promotional and educational activities targeting physicians and prescription rates of the promoted drugs have been well documented^(11)^. Some studies have shown that RD may encounter similar interactions with commercial actors^(12,13)^, which may unconsciously influence their decisions. Therefore, it is important to examine and understand the nature and potential impacts of these relationships within the dietetic profession, as lessons learned from the medical field have highlighted the significance of addressing such issues.

A recent scoping review on the interactions between nutrition professionals (including RD, a denomination used in Canada and other equivalent professions in different countries) and commercial actors (mainly food and pharmaceutical industries) identified that empirical studies on the topic were primarily published in the USA, with also studies in Spain, the UK and France, as well as in Latin America and the Caribbean region^(5)^. These interactions include gifts offered to RD by commercial actors or partnerships between professional bodies and commercial actors, among other examples^(5)^. In this scoping review, seven publications focused on Canada, of which five were non-empirical (e.g. practice paper or editorial)^(14–18)^ and two were empirical papers^(19,20)^. Among these Canadian studies, all but one discussed the risks related to these interactions with commercial actors, including the risk of undermining the credibility and trustworthiness of the nutrition profession and the risk of impairing health promotion^(14–19)^. To our knowledge, no study has focused on systematically documenting RD’ interactions with commercial actors in Canada, nor has it focused on RD’ perceptions of the topic.

In Quebec, Canada, 3392 RD were members of the professional body, the Order of Dietitians-Nutritionists of Quebec (ODNQ) on 31 March 2022^(21)^, and 441 Quebec RD were members of the professional association of the Dietitians of Canada (DC) before the end of June 2023 (information received from DC). One study published in Quebec focused on corporate political activity in the context of the Canadian food guide revision in 2020 and discussed the implications of corporate political activity for RD practice^(22)^. However, the interactions between commercial actors and RD in Quebec and Canada have yet to be studied.

At the individual level, in Quebec, Canada, RD must follow a code of ethics and meet the requirements of the ODNQ^(23)^. RD can use the code of ethics to guide decision-making around commercial actors’ engagement. However, the provisions are not specific to these relationships. For instance, the code states that an RD should preserve its independence from third-party influence without explicitly mentioning examples of third parties. More practically, in 2009, the College of Dietitians of Ontario in Canada released a professional practice paper to guide RD in identifying and managing COI, including their interactions with commercial actors^(14)^. To our knowledge, such practice papers do not exist in Quebec. At the organisational level, the ODNQ has internal policies to help manage relationships with commercial actors, and the DC also has guidelines for these relationships^(24)^. To our knowledge, these are the only mechanisms used to manage relationships between industry and RD in Quebec and Canada. However, the extent to which these mechanisms are applied and their effectiveness in protecting RD loyalty and independent judgement have not been studied.

Therefore, this study’s objective was to gain insight into the experiences and perspectives of RD working in Quebec (Canada) on their interactions with commercial actors. Specifically, in this context, this study aims to document (i) the experience and perspectives of RD about corporate political activity and COI in their professional practice and (ii) the actions undertaken by RD and their professional organisations to manage these interactions and limit their risks.

## Materials and methods

In this exploratory study, we used semi-structured interviews to document and understand RD’ experiences and perspectives about their interactions with commercial actors. We used a qualitative content analysis using both inductive and deductive methods. We triangulated and completed data collection and analysis with a document content analysis to synthesise the mechanisms to manage interactions with commercial actors.

We used a non-restrictive approach to the type of commercial actors under study, meaning that for-profit companies or corporations that produce food and drinks and third parties working for such entities, including their trade associations, public relations firms and associated scientific entities, were included.

### Semi-structured interviews

#### Sampling and recruitment

The identification and selection of participants were informed by our knowledge, professional expertise and input from an advisory committee we gathered, made up of RD working for Quebec’s dietitian’s professional bodies, the ODNQ and DC. We aimed to recruit at least one RD from five of the six main sectors of activity (where we excluded industry settings) we identified through ODNQ’s website^(25)^. Those five sectors of activity are clinical nutrition (hospital setting and private practice), public health, communication, research and education and management of food services. We aimed to capture the different contexts in which RD may work. We excluded RD currently working for commercial actors, as their obligations typically align with corporate interests rather than public health concerns. Consequently, their perspectives may be influenced by these professional obligations, as evidenced by studies examining the relationships between healthcare professionals and the pharmaceutical industry^(11,26)^. Therefore, ethical considerations regarding RD employed in commercial settings were beyond the scope of this study. We included one RD previously employed by the food industry – a relationship that ended 4 years prior to the interview (a time usually considered sufficient for the relationship not to be influential anymore^(27)^. We also aimed to recruit RD working for professional bodies and organisations from civil society known for their advocacy in nutrition policy and monitoring of corporate political activity.

Convenience and snowball sampling techniques were used to recruit participants^(28,29)^. Five participants were initially identified by the advisory committee above-mentioned and five by the research team. Then, participants interviewed were invited to identify and suggest potential key informants for our study. With the advisory committee and the research team, we completed the recruitment by identifying RD from sectors of activity that have not been included yet. The first author personally contacted twenty-one potential participants via email or private messages on LinkedIn, of which eighteen accepted our invitation. In total, three RD refused the invitation; two declined because of lack of time, and the other was not an RD anymore at the time of the study. We stopped recruiting when we achieved data saturation, defined as when we could not identify new categories, as described later, from the interviews^(30)^.

#### Interview procedures

A semi-structured interview protocol was developed by VH based on an existing interview protocol for a study of the corporate political activity of the food industry in Australia^(29)^, and it was adapted to fit our study’s objective. The interview protocol was pilot-tested with one colleague (an expert on the corporate political activity of the food industry around health professionals^(31)^) and then revised according to his and our research team’s feedback. Questions included in the interview guide can be found in the online supplementary material, Supplemental File 1. The principal investigator (VH) conducted the interviews in French (*n* 17) and English (*n* 1) using the Zoom platform from 25 May 25 to 14 August 2020. Interviews lasted, on average, 45 min and were recorded directly on Zoom, with the consent of participants. Participants had at least 1 week to read the information and consent form and send it signed by email before the scheduled interview.

#### Data analysis

Following an iterative process, we analysed data using content analysis^(32,33)^. Content analysis is well-suited for exploratory work in areas where little is known and where it aims to describe phenomena in a conceptual form^(34)^. First, VH transcribed recordings and uploaded that data using the *NVivo software* (Release 1.6.2). Second, the first author (VH) conducted a thorough reading of each transcript. Third, guided by the research objective, VH conducted the initial open coding and assigned initial codes to the data using an inductive approach: words and sentences on critical thoughts or concepts related to experiences with and perspectives about interactions with commercial actors^(32,33)^. Then, a word or a short sentence (label) from the text itself was used to name each category and sub-category.

In parallel, a deductive approach to content analysis was undertaken to guide the initial coding of the mechanisms used by RD to manage interactions with commercial actors^(32)^. Informed by a recent scoping review on existing mechanisms, we classified the mechanisms reported by participants into one of the following broader categories of mechanisms: (a) transparency, (b) management, (c) identification, (d) surveillance and (e) education and prohibition^(35)^. Lastly, the classification of data into different categories was reviewed by one of the authors (JCM). Disagreements were discussed and resolved among the team.

#### Document analysis

We conducted a document analysis to study how professional bodies, civil society organisations, government agencies (hospitals and public health organisations where RD work) and educational institutions (where RD received their initial training) have dealt with corporate political activity and COI in Quebec, Canada. We included policies, guidelines and codes of ethics that were publicly available from these organisations’ websites, as well as an internal policy and code of ethics from ODNQ. The advisory committee, as well as interviewees, facilitated the identification of the documents from these organisations.

A content analysis was conducted on these documents to identify the mechanisms that could help address and manage corporate political activity and COI. The first author read these documents to identify the type of mechanisms they covered, namely: (a) transparency; (b) management; (c) identification, surveillance and education; and (d) prohibition^(35)^. The analysis also included information on the document’s objectives and type (e.g. policy, codes or principles), the group of individuals and professional activities targeted and the presence of sanctions in the event of non-compliance.

## Results

### Participants

All participants except one were RD during the study (one was a non-RD nutrition professional working in a public health organisation advocating for nutrition policy). Among the eighteen participants, fourteen were women, and four were men. One-third of our interviewees worked in clinical nutrition (*n* 6). We also interviewed RD working in the public health nutrition sector (*n* 3), in research and education (*n* 3), in communication (*n* 2) and in management of food services (*n* 1). Three RD also worked for a professional body (DC or ODNQ).

### Summary of findings

Overall, our analysis of the semi-structured interviews highlighted that RD are experiencing various interactions with the food and pharmaceutical industries at different points in their careers. We identified six broader categories of perspectives that the interviewees discussed: (1) level of acceptability, (2) benefits, (3) risks, (4) change and evolution over time of interactions and awareness, (6) characteristics to preserve professional independence and (7) perceived barriers to address and minimise the risks associated with these interactions. This initial exploration of the issue enabled us to identify that RD and their professional organisations used several mechanisms to manage these interactions, including using the code of ethics for guidance or adopting and following policies and guidelines

### Interactions between commercial actors and registered dietitians in Quebec and conflict of interest

All participants (*n* 18) reported some interactions with the food and pharmaceutical industries throughout their careers. These interactions happened with industry trade associations, such as Dairy Farmers of Canada and the Federation of Quebec Egg Producers, and food and beverage companies and their affiliated organisations, such as Becel, Lassonde, Nestlé and Gatorade Sports Science Institute. The two pharmaceutical companies mentioned were Abbott and Bio-K.

As shown in Table 1, participants discussed five channels of interactions: (1) being exposed or invited to contribute to industry marketing, website or promotional and educational events (e.g. focus group or survey with RD led by industry), (2) interacting directly (e.g. receiving gifts and food samples), (3) receiving sponsored education, (4) interacting in a work setting (e.g. corporate lunchtime meeting) and (5) interacting through professional bodies and scientific nutrition organisations (e.g. involvement of industry in scientific and professional events). The most frequently reported interaction was the sponsorship of nutrition events and conferences (nine out of eighteen participants reported that type of interaction), promotional events and continuing education offered by commercial actors (*n* 8/18) and consulting, collaborating and being contracted to support product development (*n* 8/18). The least frequently reported interactions were jobs being offered to RD (*n* 1/18), the presence of companies’ exhibition booths in a healthcare facility (*n* 1/18) and commercial actor participation in a malnutrition project in a healthcare facility (*n* 1/18).

**Table 1:** Interactions between commercial actors and registered dietitians (RD) from Quebec

Interaction channels	Nature of interactions
Industry marketing, websites and promotional/educational events	● Promotional events and continuing education organised and offered by commercial actors (P2, P3, P4, P6, P7, P11, P16, P17) ● Focus group or survey among RD organised by commercial actors (P2, P3) ● Educational material and information developed and provided by commercial actors (P1, P11, P17, P18)
Direct interactions	● Job offers by commercial actors (P19) ● Funding of travel expenditure for training or meetings offered by commercial actors (P7, P11, P12, P17) ● Consulting, collaborating and being contracted to support product development (P2, P3, P7, P8, P9, P11, P17, P19) ● Reception of gifts, food and products samples and educational material and resources from commercial actors (P7, P9, P11, P18)
Interactions with or within educational institutions	● Collaboration/participation in commercial actors’ programmes, committees or projects (P7, P4, P14) ● Provision/sponsorship of educational materials/activities/events/internships for students offered by commercial actors (P2, P7) ● Research grant from commercial actors (P7, P14, P17)
Industry marketing/interactions in the work setting	● Employer affiliation or partnership with commercial actors (P3) ● Commercial actors exhibition booth in healthcare facility (P12) ● Attendance of lunchtime meetings (a representative from a company speaking) (P8, P12) ● Reception of commercial actors’ sponsored equipment and material and discount vouchers (P12) ● Commercial actors participation in projects about malnutrition in hospitals (P12) ● Meeting with sales representative (P6, P8, P12, P16)
Interactions through professional bodies and scientific nutrition organisations	● Involvement of commercial actors in nutrition professional and scientific events ○ Commercial actors participation in scientific event(s) (P2, P3, P4, P11, P17) ○ Exhibits booth and distribution of promotional and educational material (P8, P12, P13, P14, P18) ○ Provision of meals/beverages/food samples (P4, P17, P13) ○ Industry sponsorship of nutrition events/conference (P1, P4, P6, P7, P9, P10, P11, P13, P12, P17) ● Partnership between professional bodies and commercial actors (P2, P3, P5, P17) ● Prizes and awards sponsored by commercial actors to RD (P2, P3) ● Commercial actors advertising through association’s journal(s), direct mailing and email (P2, P5, P6, P7, P9, P12, P17)

P# = participants identification number.

### Registered dietitians’ perspectives about corporate political activity and conflict of interest in their profession

Table 2 presents key categories, sub-categories and illustrative quotes related to RD’ perspectives on their interactions with commercial actors.

**Table 2:** Key categories and illustrative quotations

Key categories	Sub-categories and illustrative quotations
Level of acceptability of interactions	Acceptable: Interactions are seen as minimal and normal *‘The underfunding of many of our training, structures and organizations can make organizations more mundane, such as the Dairy Farmers. We can forget because it’s not Coca-Cola, which is more of a bad wolf in our heads, than the DFC [Dairy Farmer of Canada], which has products that can be interesting. It’s easier to forget that they have economic interests’.* (RD working in a public health organisation) Conditional acceptability: Acceptability depends on the type of commercial actor or interaction *‘I have no problem having a company sponsor an event, but I don’t want anyone sponsoring a speaker. Do you understand? It goes to the organization and not to the speaker, whereas in other organizations, I’ve seen that… that makes me scream’. (*RD working for a professional body or nutrition association)
Perceived benefits of interactions	Providing good quality nutrition information and education for the public *‘Every time they [dairy farmers] developed material, I couldn’t help but say that it was extremely well-done educational material because it had resources that were incomparable to ours’. (*RD working in an educational institution) Improving food supply and public health *‘So, for me, it’s one of the benefits. It’s improving the food supply without the consumer realizing it and having a positive effect on public health, without even having to raise awareness among consumers’.* (RD in communication and media) Continuing education and information for professionals *‘Yes, both positive and negative influences. I see good in it. To know what’s coming on the market. (…) Otherwise, we lack information. The naturopaths come to know more, and everybody is more interested in them. Yes, there may be a lack of scientific data, but it is important to know that it exists and is coming’.* (Clinician RD) Partnerships between professional bodies and commercial actors can reduce membership cost *‘Yes, I know it’s very expensive, and there’s a lot of expenses, and if it wasn’t for that [sponsorship], our annual fee would be even more expensive. It’s just that we don’t want to promote that, but at the same time, it depends on what kind of food it is’.* (Clinician RD)
Perceived risks of interactions	Influencing (positively or negatively) message, decision-making and recommendations of RD *‘Because basically, nutritionists’ role is to inform neutrally, based on scientific data. As soon as we collaborate with a bio-food industry, our approach is tainted because we are linked in a way’.* (RD working for a professional body and nutrition association) Undermining the credibility and public trust of RD and the nutrition profession *‘In the long term, this type of association can damage the credibility of the people who make it and even the whole profession. If people start to perceive that dairy farmers and such companies pay nutritionists, it can even damage the profession’s credibility’.* (RD in communication and media) Influencing the public towards a positive perception of a company and its products and ultimately influencing food choices *‘The company is using the credibility of our profession to gain credentials and, therefore, give itself a health halo. Nutella is a great example because it’s cake icing, but in people’s minds, it’s breakfast food. In people’s minds, it’s one of the least bad things you can give in the morning. It’s normal for people that Nutella is part of breakfast. They feel that it’s not so bad. It shows how good their strategy was, that yes, there is a health aspect to Nutella’.* (RD in communication and media) Creating positive associations and credibility of industry brand(s) *‘Just the fact that PepsiCo or a company is funding an organization’s conference, even if we don’t realize it, it gives us a more positive view of the company. It’s unconscious, but I’m sure it gives us a more positive view of the company. And probably Pepsi is not there to tell us to buy Pepsi or that Pepsi is good. Did you know that Pepsi also has orange juice or apple juice? We also have 0-calorie Pepsi. I’m sure that they will present us with their healthy offer. This is their way of promoting to nutritionists’.* (RD working in a public health organisation) Neutralising criticism *‘The fact remains that when you have close ties with a large industry, it is very difficult to criticize it if you need to’.* (RD in communication and media) Compromising RD continuing education quality *‘Since the beginning of my career, every year, or almost every year, the dairy farmers have held a symposium on a different subject where researchers were invited to come and talk about different subjects. (…) I can’t believe that 500 nutritionists in Quebec have attended this training, recognized by the professional Order. In my opinion, this information is not as good as what we could get from independent people or a university’.* (RD in communication and media)
Change and evolution over time of interactions and increased awareness	*‘It was a year where there was a lot of awareness. [At] the OPDQ [Professional Order of Dietitians of Quebec] summit, there had been an effort by the [Order] around 2015 to limit its sponsorships. There were a few left. We were still putting maple products in the spotlight. It’s still sugar. But it wasn’t as bad as in past years. They had put in the camelina oil. My eyes were less bleeding than in 2005–2006 symposiums’.* (RD working in public health organisation) *‘But there were many years, 20 years ago, nutrition, and nutrition communications targeted at health professionals were so much more important, all the cereal companies had many dietitians as staff, whether it was General Mills, Kellogg’s. There was more communication and a lot more budget for communication aimed at health professionals than there is now. I think what I’m seeing now is that it’s much more directed at consumers than it is at healthcare professionals. Like they don’t need that middleman anymore’.* (RD working for a professional body or nutrition association)
Essential characteristics to preserve professional independence and ensure rigour (neutrality, integrity and critical thinking)	Neutrality *‘We hear a desire and appreciation for training with neutrality and objectivity […]. We hear it from the participants. It has value. We must hold on to it because otherwise, it is very expensive to develop training’.* (RD working in an educational institution) Critical thinking *‘Sometimes they have great things to give, but they are private companies. Their goal is to make money. You always must be critical’.* (RD working for a professional body or nutrition association) Integrity *‘Those who associate now, eventually it will be badly perceived, not only by the profession but also by the public. […] Let’s forget about the money side, but it’s like saying “how much is my integrity worth?” That’s really what it comes down to’.* (RD working in communication and media)
Perceived barriers to address and minimise the influence of industry on professional practice	Important source of income *‘For example, Dietitians of Canada cannot survive without massive sponsorship. You can, without being a member, subscribe and receive their newsletter. You will understand what I mean. It’s been about a year and a half since they adopted this formula. Before, you would receive a neutral newsletter, never any advertising. Now, you have messages from the Dietitians of Canada interspersed with advertising messages. If they don’t do that, they don’t survive. It’s very aggressive’.(*RD working in an educational institution) Competition between RD and other professionals, such as naturopaths *‘[…] because there are other professionals, like naturopaths and other therapists, that we can think of who are not subject to deontological codes. So, they use a lot of all these collaborations, the publicity. So, they take up a lot of space, while nutritionists feel a bit in the shadow of these professionals with no rules to follow. […] So, nutritionists have a hard time getting out of the game because they have a framework to respect and have visibility; everything in the industry is quite strong in terms of visibility. Sometimes, I find it difficult to balance respecting one’s professional obligations, wanting to be present with the public and being able to advertise […]’. (*RD working for a professional body or nutrition association) Lack of tools, training and guidelines *‘But you mentioned it at the beginning of the interview; one of the next steps that I see is that with the release of the [new] code of ethics, I think it would be useful to have guides, or training or a tool to help me make decisions when I am in a situation X as a professional: What could guide my decision? What aspects must I consider knowing if this partnership or this collaboration does not jeopardize my professional independence and does not undermine the public’s trust?’* (RD working for a professional body or nutrition association)

#### Level of acceptability

First, RD viewed interactions with commercial actors as ‘normal’ for different reasons and took a somewhat nuanced view of what interactions are and are not acceptable. For instance, three participants found these interactions ordinary and trivial, mainly when reflecting on food companies’ involvement in nutrition conferences and education. Moreover, one participant explained that food industry involvement in nutrition conferences in Quebec and Canada was limited (no food companies’ logo nor banner with only a separate exhibit room in DC or ODNQ conferences) when compared with the more imposing presence of food companies in nutrition conferences in the USA, such as the Food and Nutrition Conference and Expo. Two others perceived that RD found these interactions justified in the context of ‘underfunding of training, structure and organisations’. In addition, half of the interviewees considered that interactions were acceptable, depending on the nature of the interactions and the type of industry involved. For example, interactions with companies that produce minimally processed foods were considered acceptable, while interactions with companies whose portfolios mainly were made of ultra-processed products (industrial formulations of refined substances extracted and derived from foods and cosmetic additives^(36)^) were not.

#### Perceived benefits

RD think they can benefit from interacting with the industry. For instance, they often use educational material targeting the public and information provided by commercial actors. Indeed, they perceive the educational material created by companies to be of high quality and usefulness for RD, as companies have significantly greater financial resources than RD and their organisations. Other benefits mentioned include the possibility of improving the food supply and public health (such as by reducing the salt or sugar consumed at the population level after having influenced a company to reformulate its products), receiving free continuing education and information and the reduction of membership costs (when a commercial actor sponsors a professional body, then that budget is not needed from members).

#### Perceived risks

RD interviewed also raised concerns about whether these benefits outweighed the perceived risks associated with such relationships, particularly concerning how the public perceives and understands these interactions. For instance, collaborations and sponsorships were perceived as potentially undermining credibility and public trust towards RD. At the same time, participants also highlighted the potential of these interactions for influencing the public towards a positive perception of a company and its product, even if consuming those products leads to ill health. Although receiving free education provided by commercial actors was pointed out as a benefit, one RD questioned the quality of the information obtained when compared with independent providers.

Otherwise, some RD recognised the risk that interactions with commercial actors may influence the content of messages and recommendations they convene to the public. Two participants also mentioned that interactions with commercial actors might create positive associations and build the credibility of the commercial actors in the eyes of RD. These impacts on RD perception could be positive for healthy foods, such as fresh vegetable companies, or negative for ultra-processed products. According to interviewees, these potential influences could also neutralise RD’ criticisms towards some commercial actors.

#### Change and evolution over time of interactions and increased awareness

Another aspect addressed by RD was that the nature and frequency of interactions between commercial actors and RD were evolving and changing over time. On the one hand, there was a perception that there were fewer interactions with commercial actors in recent years compared with 20 years ago because commercial actors would turn their resources towards marketing directly to consumers instead of through health professionals. On the other hand, another participant expressed concern about the pharmaceutical industry’s influence on professional practices, particularly with the new right for RD to prescribe vitamins and minerals in Quebec. It was rather discussed that the evolving professional practices could lead to a potential for increased influence on professional practice. These perspectives show that strategies used by the industry might change according to the context and with the evolution of the profession. Additionally, some RD perceived an evolution in the perspectives and awareness across RD and their professional bodies on the issue. Indeed, they perceived more awareness and efforts to manage and minimise interactions with industry in professional bodies events, particularly from 2015 onwards.

#### Characteristics to preserve professional independence and perceived barriers to address and minimise the risks associated with these interactions

In the context of interactions with commercial actors, interviewees also emphasised important values and abilities put forward by RD to protect their independence, namely, neutrality, integrity and critical thinking. For instance, critical thinking was frequently mentioned as an essential asset in sorting out the information received by the industry. Although those values and abilities could help protect their professional independence, existing barriers to addressing and minimising commercial actors’ influence on professional practice were also considered. The most frequently mentioned barrier was financial consideration. Five participants explained that they or other colleagues rely on food company collaborations as a meaningful personal source of income, especially when working as an influencer on social media or for supplementing another job (e.g. self-employed clinical RD). Furthermore, two interviewees emphasised the rivalry between RD in Quebec and other practitioners, such as naturopaths or alternative nutrition therapists who operate without adhering to any code of ethics. These non-regulated professionals engage in social media platforms through partnerships with commercial actors. As a result, RD also seek a presence on social media to guarantee that they communicate science-based messages. However, to do this work on social media platforms, paid partnerships are often needed to survive financially, as mentioned above.

Additionally, underfunding of research and training by government agencies was mentioned as a perceived challenge to total independence. Three interviewees explained that commercial actors could be an interesting funding source for conferences, congresses or events while improving nutrition education accessibility by decreasing registration fees.

Finally, another barrier was the lack of tools and training on the issue and politics to guide decision-making on engaging with commercial actors. Existing mechanisms to address and manage interactions are discussed below.

### Mechanisms proposed or used to address and manage interactions with commercial actors and conflict of interest

RD interviewed discussed several mechanisms to address and manage interactions with commercial actors and COI. Table 3 presents these mechanisms according to whom these apply, either at the individual, institution/organisation level or both. In parallel, through our document analysis, we identified twelve documents that have been identified as potential guides for these institutions and RD in managing interactions with commercial actors. We classified those into six categories, namely policies (*n* 4 /12), code of ethics (*n* 2/12), educational tools (*n* 2/12), guidelines (*n* 1/12), code of conduct (*n* 1/12) and regulation document (*n* 1/12) (Table 4 and online supplementary material, Supplemental File 2). Across the twelve documents, all but one give guidance on how to manage interactions with commercial actors; eight highlight the importance of transparency when engaging with commercial actors or when managing COI; six are tools for COI identification, education and monitoring; and three propose prohibition strategies in specific contexts (see online Supplemental Material 2).

**Table 3:** Types of mechanisms used by registered dietitians, their professional bodies, civil society organisations and government agencies to manage interactions with commercial actors in Quebec, Canada

	Institution/organisation/professional body level	Individual level	Institution/organisation/professional body and individual level
Management	1,1) Adopting policies and guidelines (P1, P6, P7, P10, P12, P13)	2,1) Following the code of ethics of Quebec (P2, P4, P8) 2,2) Using selection criteria for choosing commercial actors’ partnership (P2, P7, P9, P11, P17)	3,1) Using signed agreement (P1, P9, P11, P13, P19) 3,2) Seeking external consultation and advice on conflict of interest (COI) (P2, P8, P11, P14, P17, P14)
Prohibiting, avoiding and refusing			3,3) Prohibiting, refusing, avoiding or withdrawing (P1, P2, P3, P4, P6, P7, P9, P10, P18, P19)
Transparency			3,4) Being transparent and disclosing interactions with commercial actors and related COI (P4, P5, P6, P7, P9, P11, P16)
Education			3,5) Educating and raising awareness (P2, P3, P7, P10, P14)
Monitoring and reporting	1,3) Monitoring and reporting of non-acceptable interactions with commercial actors in nutrition and scientific events or the media (P3, P6, P10)	2,3) Monitoring and reporting on a Facebook professional private group (P19)	

**Table 4:** Codes of ethics, policies and guidelines on interactions with commercial actors and conflict of interest in Quebec, Canada

Title of the document^*^	Organisation	Year of adoption/last update	Type of document (policy, guidelines or codes of ethics)
1) Code of Ethics of Dietitians of Quebec	ODNQ^†^	1981/2010	Code of ethics
2) (Policy on integrity and conflict of interest management)	ODNQ	2015/2021	Policy
3) (Policy on partnerships)	ODNQ	2015/2022	Policy
4) (Rules of procedure OPDQ – Code of ethics and professional conduct for directors)	ODNQ	2019/2020	Code of ethics
5) Private Sector Relationships: Principles and Guidelines	Dietitians of Canada (DC)	2015	Guidelines
6) Principle of professional practice	DC	2012	Education tool
7) (Diagnosis and management of a conflict of interest)	INSPQ	2014	Education tool
8) (Rules on conflict of interests)	University of Montreal	2009	Regulation
9) (Code of the Faculty of Medicine of the Université de Montréal concerning relations between its members and industry)	The University of Montreal, Faculty of Medicine	2017	Code of conduct
9) (Policy on Conflicts of Interest in Research, Creation, and Innovation at Université Laval)	Université Laval	2018	Policy
10) Regulation on Conflict of Interest	Mc Gill University	2011	Policy
11) Tri-Council Policy Statement	Canadian Institutes of Health Research, Natural Sciences and Engineering Research Council of Canada and Social Sciences and Humanities Research Council	2018	Policy

* Translation of the (title of the documents) was made by the first author.

† Order of Dietitians-Nutritionists of Quebec.

At the individual level, RD mentioned using the code of ethics of RD in Quebec to guide actions and decision-making. One of the provisions of the code of ethics of RD from Quebec states that ‘A dietitian shall safeguard his/her professional independence and shall ignore any intervention by a third party that could influence the performance of his/her professional duties to the detriment of the client’, which can be used by RD to guide the management of a situation with commercial actors^(23)^. However, as mentioned earlier, although some provisions can reassure RD that they respect the code of ethics when interacting with commercial actors, the code was considered insufficient to guide decision-making in these circumstances. Indeed, it was mentioned that more training and awareness-raising on this topic and a specific tool to help with decision-making should be implemented to complement this code of ethics. Another strategy, developed individually, was to define selection criteria to decide whether one can accept or not to collaborate with a specific company. Examples of these criteria include promoting only healthy food products (according to some standards not defined during the interviews) and choosing companies that correspond to one’s personal and professional values. None of the documents identified and analysed in the document analysis proposed such criteria, which must be worked out individually by RD.

At the institutional or organisational level, codes of ethics, policies and guidelines on how to deal with interactions with commercial actors and COI have been developed and implemented by universities, civil society organisations, government agencies, ODNQ and DC. An example of a document that is exclusive to those relationships is DC’s *Private Sector Relationships: Principles and Guidelines*, in which the organisation established its limit (what to prohibit or not) around interactions with commercial actors with statements such as ‘DC does not [e]ndorse any commercial products or services produced by third parties and aimed at the public. Its name and/or logo should not appear on any products or services’^(24)^. Another mechanism used at the organisational level consists of monitoring and reporting commercial actors’ attempts to influence RD and public health policies. For instance, one RD working for a civil society organisation reported having used this mechanism in the context of the involvement of commercial actors in scientific nutrition events by publicly calling out the COI of a speaker present at one of these events.

Another strategy applied at organisational and individual levels consists of signing an agreement when engaging with commercial actors to retain control over the content and the messages or to manage their participation in scientific events. Other actions proposed or used were seeking external consultations and pieces of advice on COI from independent organisations (e.g. RD asking ODNQ pieces of advice for particular situations), prohibition (e.g. RD refusing a specific collaboration with a company or avoiding all collaboration), transparency and disclosure (e.g. disclosure of sponsors at scientific events), as well as education and awareness-raising activities (e.g. a civil society organisation sent an awareness notice to RD about an invitation from a fast-food restaurant) (Table 4).

## Discussion

This study aimed to gain insights into the experience and perspectives of RD about interactions with commercial actors and COI in professional practice in nutrition in Quebec, Canada, as well as to capture what mechanisms RD and their related organisations use to prevent and manage these interactions. Our interviewees experienced various interactions with commercial actors through five different channels, which shows that commercial actors employ several strategies to interact with RD. This also points to the importance of preparing RD to manage these interactions in various contexts and, thus, having guidelines tailored for these channels.

RD interviewed were mainly exposed to commercial actors in scientific events and through continuing education. These results are aligned with existing research, where commercial actors’ involvement in health professionals’ education has been primarily documented in the last few years as part of studies on the corporate political activity of the food industry in different countries^(10,37)^. In the USA, health professionals, researchers and RD criticised commercial actors’ presence in dietetic professional education events for influencing the educational agenda and for the commercial bias introduced in training provided by these actors^(2,3,38)^. In our study, several participants also considered that commercial actors’ involvement in such events or other types of interaction could compromise RD’ continuing education quality (by receiving commercially biased information) and influence RDs recommendations (by being more inclined to recommend a product *v*. another – which could be negative in the case of ultra-processed products, for example). Despite these risks, some interviewees still felt that information and free training provided by commercial actors could be relevant, as reported by RD from other countries^(5)^. This perception has also been reported in other health professions, such as nursing in the USA, where some nurses considered that information provided by commercial actors was necessary for their practice^(39)^. However, as discussed above, education and information provided by commercial actors are often biased and do not offer counterbalancing and independent views and facts^(2,11)^.

Our interviews also highlighted that RD from Quebec have various and quite nuanced perspectives about the acceptability of these interactions and COI. Some participants considered interactions in Quebec not concerning (especially compared with the USA), while others worried that these relationships were trivialised among RD. Interestingly, dietetic professionals reported similar views in France^(26)^. The reluctance to criticise the interactions with commercial actors in France would come from a fear of creating antagonism between professionals, or in other words, creating ‘(confusion among health professionals, that considered themselves as more credible if they speak as one voice)’ (translation by the authors)^(26)^. For other RD in our interviews, acceptability was conditional and depended primarily on the type of interactions and commercial actors being involved. Lastly, there was also a group of RD that avoided all interactions with commercial actors. These divergent perspectives can be compared with a typology developed in Australia that defined three profiles of physicians depending on how they described their interactions with the pharmaceutical industry: (1) ‘Avoiders, who tend to avoid direct contacts with industry, because it was a synonym of promoting it and that there was always an intention to influence their prescriptions’; (2) ‘Ambivalent engagers, who engaged with a certain level of reluctance and are afraid that interactions could compromise their autonomy and create a COI, but get involved with it anyway’; and (3) ‘Confident engagers, who engaged actively with industry because these interactions were beneficial and these contacts were an opportunity to share, which seems for them beneficial for patients’^(40,41)^. Interestingly, we could also distinguish these three profiles among our participants within the discussion about their experiences with commercial actors and their perspectives on the potential impacts on professional practice.

We found that some RD, who could be described as ‘ambivalent engagers’, would perceive barriers to minimising interactions with commercial actors and managing these relationships. They express concerns about competing with non-health professionals, suggesting that paid collaborations could boost their visibility on social media, countering messages from these non-professional health advisers. Despite acknowledging potential risks to their reputation, they believed maintaining some relationships with commercial actors, particularly on social media, can secure income for professional activities (such as educating about healthy eating) and enhance visibility. Additionally, there was a perception that there were fewer interactions with commercial actors in recent years than 20 years ago, which was explained by a change in the marketing strategies mainly directed at consumers. Using high-profile but unregulated non-professional health advisers or influencers might be one of these new marketing strategies, similar to the pharmaceutical industry shift in marketing focus made in recent years. Indeed, this industry would rely more on digital advertising and engagement tactics for direct-to-consumer marketing^(42)^.

Nevertheless, some RD felt these relationships might jeopardise one’s and the profession’s credibility, one of the more important risks discussed that pertains to public trust. In Quebec, public trust and perception are critical to the nutrition profession, and RD still often must debate and demonstrate the value of their contribution to society and public health to other health professionals and the public. Ensuring that the public recognises and trusts RD as leading experts in nutrition can help protect the public from nutrition disinformation^(43)^. Thus, participants highlighted the importance of critical thinking, neutrality and integrity to maintain their credibility and achieve their professional responsibility towards corporate political activity and COI. During dietetic training in France, the significance of cultivating critical thinking skills among dietitians and students was also emphasised, enabling them to recognise and evaluate influential strategies, identify key actors and the interests they defend in their field of practice and prioritise the well-being of patients and the public they serve^(26)^. Education and awareness-raising are fundamental mechanisms that could help address the risks associated with corporate political activity and COI. RD interviewed in our study also discussed these mechanisms. RD would often refer to the code of ethics, their professional body (i.e. ODNQ) or even colleagues in case of doubt on managing these interactions. Since the code of ethics was not explicitly developed for these interactions and COI management, we also identified a desire across RD interviewed for more standardised and clear guidelines, ideally suited to each of their sectors of activity, aside from the code of ethics.

Furthermore, some participants had personal criteria for engaging with the commercial actors based on the nutritional value of a company’s product and alignment with their values. In 2019, a scoping review on the interactions between health researchers and the food industry emphasised the importance of ensuring compatibility between researchers’ goals or values and those of food companies^(44)^. For instance, one of the principles identified in the review was to avoid engaging with companies ‘whose objectives and/or goals are related to the increased production, supply or demand of “unhealthy food” products and/or to the promotion of unhealthy and unsustainable ways of eating and producing food’^(44)^. However, the definition of ‘unhealthy food’ can vary from one RD or organisation to another, resulting in RD going in all directions with these guidelines. One potential solution could be using the Canadian food guide to define what is ‘healthy or not’^(45)^. Nevertheless, if the healthiness of a specific food product is to be included in such guidelines, it should not be the only criterion for deciding whether to engage with a food company. Additional information, such as corporate practices that impact health and equity, should also be taken into consideration, including, for instance, tax evasion, lobbying against public health policies and treatment of employees^(46)^.

Finally, the three universities that offer the initial training of RD in Quebec have a COI interest policy or rule within their institution. However, only the University of Montreal has a specific code for relationships between commercial actors and the Faculty of Medicine’s members, which include the Department of Nutrition^(47)^. To our knowledge, McGill University and University Laval only hold a broader policy on COI that is not specific to the interactions with the food and pharmaceutical industries.

### Implication for research and professional practice

There is a need for more systematic research on COI and corporate political activity targeting RD beyond Quebec and Canada to better capture the nature and the extent of these interactions. These findings may also stimulate further discussions about these issues within the profession while simultaneously bringing critical evaluation of RD’ interactions with commercial actors and their impacts.

Despite policies and solutions addressing COI and their influence on RD exist, there is limited evidence regarding their effectiveness. These are also insufficient to prevent or manage COI and protect RD professional independence. Recently, new guidelines on COI management in public health nutrition have been launched by the WHO and the UNICEF^(48,49)^. The WHO drafted a six-step tool to help with decision-making around collaboration with commercial actors in nutrition programmes for state members^(48)^ and published guidance on sponsoring health professional and scientific meetings by companies that market foods for infants and young children^(50)^. These examples could serve as a starting point for developing more specific guidelines for RD and their professional bodies to help with decision-making in this area.

### Strengths and limitations

This study has limitations and strengths. It is the first study in Quebec focusing on RD’ experience and perceptions in interacting with commercial actors. We covered different sectors of activity in nutrition, and our sample had quite a rich diversity of interviewees. However, some perspectives may be unrepresented in our study. For instance, RD in the clinical sector of activity from different work settings (e.g. home support or family medicine group) than the work settings of the RD interviewed in this study (e.g. private practice or hospital) might have had different experiences with the industry and other strategies to manage those interactions. Finally, we did not require participants to fill out COI declaration forms before the interview, which could have revealed their potential bias. However, this kind of practice can sometimes represent a barrier to participation for those who have COI.

### Conclusion

We unveiled the existing relationships between RD and commercial actors in Quebec, Canada. We identified that RD experience diverse interactions with commercial actors and have different perceptions about the potential benefits and risks associated with such interactions. Mechanisms exist to manage (or prevent, where relevant) these interactions, but little is known about their effectiveness. Awareness-raising activities and training are needed to safeguard the credibility and public trust in RD, who are important actors in public health protection and promotion.

## Supporting information

Hamel et al. supplementary material Hamel et al. supplementary material

## Supplementary material

For supplementary material accompanying this paper, visit https://doi.org/10.1017/S1368980024001733.

## References

[1] Rodwin MA (2018) Attempts to redefine conflicts of interest. Account Res 25, 67–78. 29172685 10.1080/08989621.2017.1405728

[2] Simon M (2013) Are America’s Nutrition Professionals in the Pocket of Big Food? Eat Drink Politics. Oakland, CA: Eat Drink Politics.

[3] Nestle M (2013) Food Politics: How the Food Industry Influences Nutrition and Health, 3rd ed. (Berkley and Los Angeles), California: University of California Press.

[4] Simon M (2015) Is the Dietitians Association of Australia in the Pocket of Big Food? Oakland, CA: Eat Drink Politics.

[5] Hamel V , Hennessy M , Mialon M et al. (2023) Interactions between nutrition professionals and industry: a scoping review. Int J Health Policy Manage 12, 1–17. 10.34172/ijhpm.2023.7626 PMC10590255 38618820

[6] Ulucanlar S , Lauber K , Fabbri A et al. (2023) Corporate political activity: taxonomies and model of corporate influence on public policy. Int J Health Policy Manage 12, 1–22. 10.34172/ijhpm.2023.7292 PMC10462073 37579378

[7] Newton A , Lloyd-Williams F , Bromley H et al. (2016) Food for thought? Potential conflicts of interest in academic experts advising government and charities on dietary policies. BMC Public Health 16, 735. 27495802 10.1186/s12889-016-3393-2 PMC4975877

[8] Richards Z , Thomas SL , Randle M et al. (2015) Corporate social responsibility programs of Big Food in Australia: a content analysis of industry documents. Aust N Z J Public Health 39, 550–556. 26259972 10.1111/1753-6405.12429

[9] Pereira TN , Nascimento FA & Bandoni DH (2016) Conflict of interest in the training and practices of nutritionists: regulation is necessary. Cien Saude Colet 21, 3833–3844. 27925123 10.1590/1413-812320152112.13012015

[10] Mialon M , Jaramillo A , Caro P et al. (2020) Involvement of the food industry in nutrition conferences in Latin America and the Caribbean. Public Health Nutr 24, 1559–1565. 10.1017/S1368980020003870 PMC10195453 33118920

[11] World Health Organization & Health Action International (2009) Understanding and Responding to Pharmaceutical Promotion. https://www3.paho.org/hq/dmdocuments/2011/drug-promotion-manual-CAP-3-090610.pdf (accessed July 2023).

[12] Grundy Q , Bero L & Malone R (2013) Interactions between non-physician clinicians and industry: a systematic review. PLoS Med 10, e1001561. 24302892 10.1371/journal.pmed.1001561 PMC3841103

[13] McInnes RJ , Wright C , Haq S et al. (2007) Who’s keeping the code? Compliance with the international code for the marketing of breast-milk substitutes in Greater Glasgow. Public Health Nutr 10, 719–725. 17381952 10.1017/S1368980007441453

[14] Cohen D (2009) Conflict of Interest & RD Practice. Toronto, ON: College of Dietitians of Ontario.

[15] Freedhoff Y & Hebert PC (2011) Partnerships between health organizations and the food industry risk derailing public health nutrition. CMAJ 183, 291–292. 21282309 10.1503/cmaj.110085 PMC3042434

[16] Freedhoff Y (2014) The food industry is neither friend, nor foe, nor partner. Obes Rev 15, 6–8. 24330345 10.1111/obr.12128

[17] Gingras J (2005) Evoking trust in the nutrition counselor: why should we be trusted? J Agric Environ Ethics 18, 57–74.

[18] Gramlich L (2011) Health organizations and the food industry. CMAJ 183, 934. 10.1503/cmaj.111-2048 PMC3091907 21576316

[19] Potvin Kent M , Pauzé E , Guo K et al. (2020) The physical activity and nutrition-related corporate social responsibility initiatives of food and beverage companies in Canada and implications for public health. BMC Public Health 20, 890–890. 32517669 10.1186/s12889-020-09030-8 PMC7281932

[20] Olstad DL , Raine KD & McCargar LJ (2013) The role of registered dietitians in health promotion. Can J Dietetic Pract Res 74, 80–83. 10.3148/74.2.2013.80 23750980

[21] Ordre des diététiste nutritionnistes du Québec (2022) Annual Report 2021–2022. Quebec, Canada. https://odnq.org/wp-content/uploads/2022/10/Rapport_annuel_OPDQ_2021-2022.pdf (accessed July 2023).

[22] Robitaille M-C , Hamel V & Moubarac J-C (2020) Corporate political activity and influence on public policy: an important issue for public health nutrition. Nutr science en évolution 18, 14–23.

[23] Quebec Official Publisher (2022) Code of Ethics of Dietitians, Vol. RLRQ c. C-26, r.97, Art. 20. Quebec City, QC, Canada: LégisQuébec.

[24] Dietitians of Canada (2015) Private Sector Relationships: Principles and Guidelines. https://www.dietitians.ca/DietitiansOfCanada/media/Documents/Resources/Private-Sector-Relationships-Principles-and-Guidelines_Aug2015.pdf (retreived - accessed August 2023).

[25] Ordre des diététiste nutritionnistes du Québec (2023) Sectors of activity. https://odnq.org/grand-public/secteurs-dactivite/ (accessed 20 June 2023).

[26] Scheffer P (2015) Healthcare professions and the pharmaceutical, agri-food and chemical industries: What critical training course? [L’Harmattan, editor]. Paris: L’Harmattan, 171–176.

[27] OECD/PRI (2022) Regulating Corporate Political Engagement: Trends, Challenges and the Role for Investors. Paris: OECD/PRI.

[28] Cullerton K , Donnet T , Lee A et al. (2016) Playing the policy game: a review of the barriers to and enablers of nutrition policy change. Public Health Nutr 19, 2643–2653. 27034196 10.1017/S1368980016000677 PMC10271040

[29] Mialon M , Swinburn B , Allender S et al. (2017) ‘Maximising shareholder value’: a detailed insight into the corporate political activity of the Australian food industry. Aust N Z J Public Health 41, 165–171. 28110500 10.1111/1753-6405.12639

[30] Saunders B , Sim J , Kingstone T et al. (2018) Saturation in qualitative research: exploring its conceptualization and operationalization. Qual Quant 52, 1893–1907. 29937585 10.1007/s11135-017-0574-8 PMC5993836

[31] Scheffer P Dietitian training and critical thinking: How can we foster professional independence and reflective practice? France: L’Harmattan.

[32] Hsieh HF & Shannon SE (2005) Three approaches to qualitative content analysis. Qual Health Res 15, 1277–1288. 16204405 10.1177/1049732305276687

[33] Neuman WL (2011) Social Research Methods: Qualitative and Quantitative Approaches, 7th ed. Boston: Pearson.

[34] Vaismoradi M , Turunen H & Bondas T (2013) Content analysis and thematic analysis: implications for conducting a qualitative descriptive study. Nurs Health Sci 15, 398–405. 23480423 10.1111/nhs.12048

[35] Mialon M , Vandevijvere S , Carriedo-Lutzenkirchen A et al. (2020) Mechanisms for addressing and managing the influence of corporations on public health policy, research and practice: a scoping review. BMJ Open 10, e034082. 10.1136/bmjopen-2019-034082 PMC7371213 32690498

[36] Monteiro CA , Cannon G , Levy RB et al. (2019) Ultra-processed foods: what they are and how to identify them. Public Health Nutr 22, 936–941. 30744710 10.1017/S1368980018003762 PMC10260459

[37] Mialon M & Mialon J (2018) Analysis of corporate political activity strategies of the food industry: evidence from France. Public Health Nutr 21, 3407–3421. 29998811 10.1017/S1368980018001763 PMC10260908

[38] Dietitians for Professional Integrity (2013) The Food Ties that Bind: The Academy of Nutrition & Dietetic’ 2013 Conference & Expo. http://smallbites.andybellatti.com/wp-content/uploads/2013/11/The-Food-Ties-that-Bind-Nov-18.pdf (accessed July 2023).

[39] Grundy Q , Bero LA & Malone RE (2016) Marketing and the most trusted profession: the invisible interactions between registered nurses and industry. Ann Intern Med 164, 733–739. 27043976 10.7326/M15-2522

[40] Doran E , Kerridge I , McNeill P et al. (2006) Empirical uncertainty and moral contest: a qualitative analysis of the relationship between medical specialists and the pharmaceutical industry in Australia. Soc Sci Med 62, 1510–1519. 16143441 10.1016/j.socscimed.2005.07.037

[41] Grenouilleau, A-S (2022) Interactions of health professionals with industry representatives. France: Haute Autorité de Santé.

[42] Willis E & Delbaere M (2022) Patient influencers: the next frontier in direct-to-consumer pharmaceutical marketing. J Med Internet Res 24, e29422. 35230241 10.2196/29422 PMC8924782

[43] Ordre des diététiste nutritionnistes du Québec (2024) What is the Order doing to protect me? https://odnq.org/grand-public/que-fait-lordre-pour-me-proteger/ (accessed 25 April 2024).

[44] Cullerton K , Adams J , Forouhi N et al. (2019) What principles should guide interactions between population health researchers and the food industry? Systematic scoping review of peer-reviewed and grey literature. Obes Rev 20, 1073–1084. 30968553 10.1111/obr.12851 PMC6767600

[45] Government of Canada (2019) Canada’s Food Guide, Limit Highly Processed Foods. Canada: Government of Canada.

[46] Gilmore AB , Fabbri A , Baum F et al. (2023) Defining and conceptualising the commercial determinants of health. Lancet 401, 1194–1213. 36966782 10.1016/S0140-6736(23)00013-2

[47] Faculty of medicine - University of Montreal (2017) Code on relations between members of the Faculty of medicine and industry. https://ethiqueclinique.umontreal.ca/wp-content/uploads/sites/14/2018/01/code-ethique-industrie.pdf (accessed July 2023).

[48] World Health Organization (2017) Safeguarding against Possible Conflicts of Interest in Nutrition Programmes. https://apps.who.int/iris/bitstream/handle/10665/274165/B142_23-en.pdf?sequence=1&isAllowed=y (accessed August 2023).

[49] United Nations Children’s Fund (UNICEF) (2023) Engaging with the Food and Beverage Industry. UNICEF Programme Guidance. https://www.unicef.org/media/142056/file/Programme%20Guidance%20on%20Engagement%20with%20the%20Food%20and%20Beverage%20Industry.pdf (accessed August 2023).

[50] World Health Organization (2023) Clarification on Sponsorship of Health Professional and Scientific Meetings by Companies that Market Foods for Infants and Young Children: Information Note. https://apps.who.int/iris/bitstream/handle/10665/369451/9789240074422-eng.pdf (accessed August 2023).

