# Supplementary material for: ‘The company is using the credibility of our profession’: exploring experiences and perspectives of registered dietitians from Canada about their interactions with commercial actors using semi-structured interviews
Source: Public Health Nutr. 2024 Oct 4;27(1):e196. doi: 10.1017/S1368980024001733 (PMC11504647; doi:10.1017/S1368980024001733)
Supplement: Hamel et al. supplementary material [file S1368980024001733sup001.docx]

## Supplementary Material 2. Codes of ethics, policies, and guidelines on interactions with commercial actors and COI in Quebec, Canada

| **Title of the document** | **Organization** | **Year of adoption/ Last update** | **Type of document (policy, guidelines, Code)** | **Objective(s)** | **Type of mechanism** | **Group of individuals targeted by the document** | **Professional activities targeted by the document** | **Sanction in case of non-compliance (Yes/no)** |
| --- | --- | --- | --- | --- | --- | --- | --- | --- |
| 1. Code of Ethics of Dietitians (free translation) | ODNQ* | 1981/2010 (2021)** | Code of Ethics | To protect the public. | - Management - Prohibition | - Registered dietitians, members of ODNQ | Professional practice in all sectors of activity | Yes |
| 1. Policy on integrity and conflict of interest management (free translation) | ODNQ | 2015/2021 | Policy | To value integrity, ensure the respect and promotion of the values of ethics and integrity for the ODNQ’s activities, preserve the trust of the public, the ODNQ’s members and partners, provide mechanisms to identify, manage and prevent situations of conflict of interest and disclosure of interest, ensure that the ODNQ, its bodies, the members of its committees, and the speakers and trainers engaged take adequate measures to avoid, reduce or manage conflicts of interest (free translation). | - Management - Transparency | - Administrative members of ODNQ - Lecturers, trainers, and partners of ODNQ | ODNQ activities | Yes |
| 1. Policy on partnerships (free translation) | ODNQ | 2015/2022 | Policy | To guide ODNQ's actions concerning partnerships, which are intended to support ODNQ’s activities by following its mission and values. To formalize the commitments and responsibilities of the partners when establishing a partnership (free translation). | - Management - Transparency | - Administrative members of ODNQ - Lecturers, trainers, and partners of ODNQ | ODNQ’s engagement with external partners | N/A |
| 1. Rules of procedure OPDQ - Code of ethics and professional conduct for directors (free translation) | ODNQ | 2019/2020 | Code of Ethics | Preserve and strengthen the bond of trust between the public and the members of the ODNQ and the administration of the ODNQ to promote transparency within the ODNQ, to make the members of its Board of Directors accountable for ethical and deontological issues and to raise awareness of the general management of the Order (free translation). | - Management - Transparency - Identification, education, and monitoring | - Administrative members of ODNQ | Exercise of the functions of the Board of Directors of the ODNQ | Yes |
| 1. Private Sector Relationships: Principles and Guidelines | Dietitians of Canada (DC) | 2015 | Guidelines | Not stated | - Management - Prohibition - Transparency | - Dietitians of Canada | Interactions between Dietitians of Canada and the private sector, service to members of Dietitians of Canada, and public services | No |
| 1. Principle of professional practice | Dietitians of Canada (DC) | 2012 | Education tool | Not stated | - Identification, education, and monitoring | - Registered dietitians, members of Dietitians of Canada | Professional practice in all activity sectors | N/A |
| 1. Diagnosis and management of a conflict of interests (free translation) | INSPQ | 2014 | Education tool | To help with decision-making when choosing a partner for a project about food and physical activity (free translation). | - Management - Identification, education, and monitoring | - Public health and nutrition organizations | Partnerships/interactions between public health and nutrition organizations and partners, such as industry | N/A |
| 1. Rules on conflict of interests (free translation) | University of Montreal | 2009 | Regulation | To prevent conflict of interests and protect individuals and the institution (free translation). | - Management - Transparency, Identification, education, and monitoring | - Employees of the University of Montreal - Students implied in research at the University of Montreal | Education and research | Yes |
| 1. Code of the Faculty of Medicine of the Université de Montréal concerning relations between its members and industry (free translation) | The University of Montreal, Faculty of Medicine | 2017 | Code of conduct | To sets out its members' relationships with the industry during their activities. Enrich the critical thinking of everyone (free translation). | - Management - Transparency - Prohibition - Identification, education, and monitoring | - Employees of the University of Montreal - Students implied in research at the University of Montreal | Awards and grants, educational activity, research, management of industry funds, industry-funded activities, gifts, consulting contracts and services, access by industry representatives, samples, equipment donations and loans, clinical decisions, purchase or lease decisions, management of conflicts of interest | No |
| 1. Policy on Conflicts of Interest in Research, Creation and Innovation at Université Laval (free translation) | Université Laval | 2018 | Policy | - Identify relationships that may lead to a conflict of interest.  - To define what constitutes a conflict of interest in research and creation.  - Provide a mechanism for disclosing a conflict of interest and, when it cannot be avoided, for resolving it responsibly  to resolve it responsibly.  - To provide guidance to members of the University so that they do not find themselves in a conflict-of-interest  when it could have been avoided or resolved in a transparent and impartial manner (free translation). | - Management - Transparency - Identification, education, and monitoring | - All research actors at Université Laval | Research | Yes |
| 1. Regulation on Conflict of Interest | Mc Gill University | 2011 | Policy | “The purposes of this Regulation are to ensure that:  - all affairs of the University are conducted in a manner that is free of actual and apparent conflict of interest and maintains the trust of the community in the University and its affiliated institutions;  - at all times, all members of the University Community act with integrity and adhere to the highest ethical standards;  - the integrity of all members is protected in the performance of their University’s obligations and functions;  - all members can identify actual or apparent conflicts of interest; and  - all actual and apparent conflicts of interest are properly managed in keeping with the law and best practices. | - Management | - Employees of McGill University - Members who hold office under the University Charter or Statutes or who serve on any body or committee of the University - Members who hold office on the board of an institution affiliated with McGill University or who serve on a committee established by such board - Appointees (including a volunteer) of the University | Education and research | Yes |
| 1. Tri-Council Policy Statement | Canadian Institutes of Health Research, Natural Sciences and Engineering Research Council of Canada, and Social Sciences and Humanities Research Council | 2018 | Policy | Chapter 7. Conflicts of Interest: Do not compromise the integrity of the research and protect participants. | - Management - Transparency | - Academia | Research | Yes |

*Ordre des Diététistes-Nutritionnistes du Québec

** Note: the Code was modified only because the name of the professional body changed in 2021; it is still the same version of the one from 2010.
